# Alterations in urine, serum and brain metabolomic profiles exhibit sexual dimorphism during malaria disease progression

**DOI:** 10.1186/1475-2875-9-110

**Published:** 2010-04-23

**Authors:** Angika Basant, Mayuri Rege, Shobhona Sharma, Haripalsingh M Sonawat

**Affiliations:** 1 Department of Biological Sciences, Tata Institute of Fundamental Research, Homi Bhabha Road, Mumbai - 400 005, India; 2 Department of Chemical Sciences, Tata Institute of Fundamental Research, Homi Bhabha Road, Mumbai - 400 005, India; 3 Program in Molecular Medicine, Suite 203, Two Biotech, 373 Plantation St, Worcester, MA 01605, USA

## Abstract

**Background:**

Metabolic changes in the host in response to *Plasmodium *infection play a crucial role in the pathogenesis of malaria. Alterations in metabolism of male and female mice infected with *Plasmodium berghei *ANKA are reported here.

**Methods:**

^1^H NMR spectra of urine, sera and brain extracts of these mice were analysed over disease progression using Principle Component Analysis and Orthogonal Partial Least Square Discriminant Analysis.

**Results:**

Analyses of overall changes in urinary profiles during disease progression demonstrate that females show a significant early post-infection shift in metabolism as compared to males. In contrast, serum profiles of female mice remain unaltered in the early infection stages; whereas that of the male mice changed. Brain metabolite profiles do not show global changes in the early stages of infection in either sex. By the late stages urine, serum and brain profiles of both sexes are severely affected. Analyses of individual metabolites show significant increase in lactate, alanine and lysine, kynurenic acid and quinolinic acid in sera of both males and females at this stage. Early changes in female urine are marked by an increase of ureidopropionate, lowering of carnitine and transient enhancement of asparagine and dimethylglycine. Several metabolites when analysed individually in sera and brain reveal significant changes in their levels in the early phase of infection mainly in female mice. Asparagine and dimethylglycine levels decrease and quinolinic acid increases early in sera of infected females. In brain extracts of females, an early rise in levels is also observed for lactate, alanine and glycerol, kynurenic acid, ureidopropionate and 2-hydroxy-2-methylbutyrate.

**Conclusions:**

These results suggest that *P. berghei *infection leads to impairment of glycolysis, lipid metabolism, metabolism of tryptophan and degradation of uracil. Characterization of early changes along these pathways may be crucial for prognosis and better disease management. Additionally, the distinct sexual dimorphism exhibited in these responses has a bearing on the understanding of the pathophysiology of malaria.

## Background

*Plasmodium *is the causative organism of malaria which affects 200-300 million people and causes nearly one million deaths annually [1]. This poses a serious, global health problem. The clinical symptoms of this parasitic infection are seen during the blood stages [2].

During the acute stages of the disease more than one tissue type of the host is known to be affected [2,3]. In severe cases of *Plasmodium falciparum *infection, parasitized red cells are seen to exhibit sequestration, which in turn affect the microvasculature in various tissues such as heart, eyes, liver, kidneys, intestines, and adipose tissue [4-6] possibly resulting in localized metabolic stress. As the disease approaches late stages, several complications accumulate possibly through inflammatory immune responses and manifest themselves into one or more conditions, such as liver damage, cerebral malaria, renal damage, severe anaemia, hypoglycaemia and acidosis, which are often causes of death [7-9]. The transition into these conditions is incompletely understood and there are no reliable predictors for them presently. However, all these manifestations of the disease are associated with drastic metabolic alterations in the host. Therefore, metabolites in body fluids and tissues that correlate with progression of the disease may serve as useful indicators of these changes. These metabolites may underscore the metabolic pathways that are perturbed in the host in response to infection.

^1^H NMR spectroscopy of tissues and bio-fluids followed by multivariate analyses of the spectroscopic data is a systems biological approach that has been used to identify important changes in metabolism [10]. ^1^H NMR spectra of such samples provide an unbiased profile of all metabolites. The strategy has been used to study various disease conditions including breast cancer, diabetes, coronary heart disease and high blood pressure [11-14]. Metabolic responses of living systems to genetic modification, environmental changes and drug-toxicity have also been studied in various animal models with this approach [15,16]. Furthermore, parasitic infections, such as that induced by *trypanosomes *and *schistosomes*, have been investigated in mouse models and relevant metabolites linked with the disease have been identified [17-20].

Composition of bio-fluids and tissues reflects the metabolic status of a living system. In the study here adult male and female BALB/c mice were infected with *Plasmodium berghei *and ^1^H NMR spectra of urine samples, sera and whole brain extracts were analysed. This is an experimental model of non-cerebral malaria (NCM), identical to the one used in a previous study [3]. Host metabolic alterations have been characterized in this system. The experiments using urine samples helped to non-invasively track the same animals over the progression of the disease. The sera and brain extracts were useful in further characterizing the early and late phase metabolic changes during malaria. All the spectra were analysed using Principal Component Analysis (PCA) and Orthogonal Partial Least Square-Discriminant Analysis (OPLS-DA) algorithms to elucidate the identity of metabolites that change with the progress of the disease. The results of these experiments are presented here. It is clearly demonstrated that the response of male and female mice to *Plasmodium *infection is different in its temporal nature and degree. In addition, the metabolites perturbed in the urine, serum and brain exhibit distinct sex-specific dimorphism. This would have implications in the pathophysiological susceptibility differences of males and females to malaria.

## Methods

### Animal experiments

The animals used in the study were treated in accordance with the guidelines of the Local Animal Ethics Committee of TIFR.

### Urine sample collection

**Figure 1:**
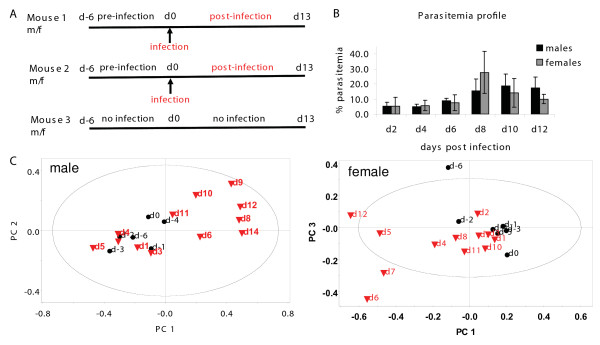
**Mouse experiments with malarial infection: experiment, parasitaemia and ^1^H NMR based PCA of urine**. A. Each experiment consisted of 3 males (m) and 3 females (f) BALB/c mice of the same litter, housed in individual metabolic cages. The mice were daily sampled for urine for 20 days (13 days post-infection). The arrow indicates the day of infection with *Plasmodium berghei ANKA*. B. The average parasitaemia profile of the mice as a function of disease progression. C. A representative scores plot showing the trajectory of a single infected animal. Each point corresponds to the day indicated. Black circle = pre-infection, Red triangle = post-infection. The ellipse is a 95% Hotelling's T^2 ^ellipse. R^2^X (cum) = 0.794 (male), 0.829 (female).

Eighteen inbred BALB/c mice; aged 6 to 8 weeks were used. The experiments were conducted in three batches. Each batch had three male and three female mice belonging to the same litter. Each mouse was housed individually in a metabolic cage (Tecniplast, Italy) that was suitably modified for BALB/c mice. The mice were kept under stable environmental conditions (12-hour day-night cycle, 22 ± 2°C) with free access to water and standard food pellets. Mice were acclimatized to the cages for seven days. In two of the three batches, on the 7^th ^day, two male and two female mice of the litter were injected intra-peritoneally with 10^6 ^*P. berghei *(ANKA strain) infected erythrocytes. All six mice were monitored for 12-14 days post-infection. In the third batch, all six mice were housed in the cages for the entire duration without infection. Each day a 24-hour urine sample for each mouse was collected from the collecting tube at the bottom of the metabolic cage between 1500 and 1700 hours. Cages were cleaned daily to avoid contamination in the next sample. The collecting tube contained 100 μL of 1% sodium azide to prevent microbial growth and samples were stored at -20°C as described below. Such a procedure would average out diurnal variations and stress related transient changes in the urine composition. Since animals used for comparison were littermates and were subjected to the same diet and laboratory environment, minimal metabolic differences over the period of 21 days were expected for control uninfected mice of the same sex. Also when mice of the same sex were compared before and after infection, it was presumed the changes observed would be solely due to the disease. Parasitaemia was monitored in the infected mice by taking tail bleeds, and examining Giemsa-stained blood-smears [21]. The schematics of the experimental paradigm and the progress in parasitaemia in the experiments are shown in Figure (1A and 1B). Beyond day 13 post-infection, the parasitaemia rises beyond 30% and mice died after 4-6 days.

### Serum and brain sample collection

**Figure 2:**
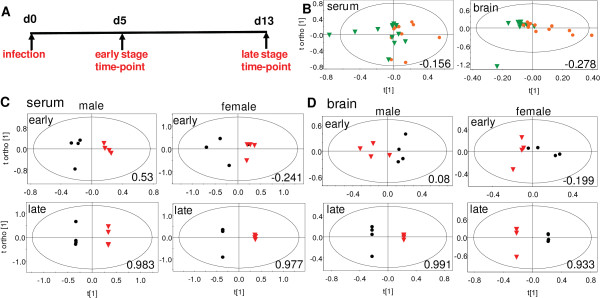
**Mouse experiments with malarial infection: experiment and ^1^H NMR based OPLS-DA of serum and brain**. A. The experiment consisted of 12 males and 12 females. The parasite *P. berghei ANKA *was injected in 8 males and 8 females on day 0. On day 5 post-infection, 4 uninfected controls (2 males, 2 females) and 8 infected animals (4 males, 4 females) were sacrificed and whole brain tissue and serum collected. The remaining animals were sacrificed similarly on day 13 post-infection. B. Scores plot of the OPLS-DA model of serum and brain from all 24 animals showing male-female class separation. R^2^X(cum), Q^2^(cum) = 0.506, -0.156 serum; 0.564, -0.278 brain. Temporal metabolic changes in C. serum and D. brain have been shown by comparing day 5 (early) and day 13 (late) post-infection with uninfected controls. C. R^2^X(cum), Q^2^(cum) = 0.87, 0.531 males, early; 0.994, 0.983 males, late; 0.744, -0.241 females, early; 0.996, 0.977 females, late D. R^2^X(cum), Q^2^(cum) = 0.554, 0.08 males, early; 0.981, 0.991 males, late; 0.698, -0.199 females, early; 0.985, 0.933 females, late.

Twenty-four inbred BALB/c mice (12 male, 12 female) aged 6 to 8 weeks were used. Eight male and eight female mice were injected intra-peritoneally with 10^6 ^infected erythrocytes. On day 5 post-infection, eight infected mice (four male, four female, %parasitaemia = 2.45 ± 0.70 males, 1.53 ± 0.35 females) and four uninfected mice (two male, two female) were sacrificed by placing them in an ether chamber. The animals were immediately dissected. Blood was collected into Eppendorf tubes directly by heart puncture and allowed to clot. Also, the whole brain was extracted, snap-frozen in liquid nitrogen and stored at -80°C until further processing. This procedure was repeated for the remaining animals on day 13 post-infection (%parasitaemia = 27.03 ± 5.57 males, 27.13 ± 10.76 females). The experimental scheme is shown in Figure 2A.

### Sample preparation

#### Urine

Samples were mixed with 0.2 M phosphate buffer (pH 7.4) to make the volume ~750 μL and centrifuged. The supernatant was stored at -20°C until further use for NMR spectroscopy. To prepare samples for NMR experiments 40 μL of 0.12% 2, 2-dimethyl-2-silapentane-5-sulfonic acid (DSS) in D_2_O was added to 460 μL of the urine sample such that DSS (for chemical shift reference) was present to the extent of 0.01% in the NMR sample.

#### Serum

Fresh blood samples were incubated at 37°C for 10 minutes and subsequently centrifuged at 13,100 g for 10 minutes. Perchloric acid was added (final concentration 5%) to the supernatant. The samples were centrifuged at 11,300 g at 4°C. The supernatants were neutralized using 50% KOH and recentrifuged at 11,300 g. The residues were discarded and the supernatants were dried in a speedvac and stored at -20°C. The samples were reconstituted for NMR experiments in D_2_O containing DSS at 0.01%.

#### Brain

Each whole brain was thawed and homogenized in phosphate buffer saline (137 mM NaCl, 2.7 mM KCl, 4.3 mM Na_2_HPO_4_, 1.47 mM KH_2_PO_4_, pH 7.4) using a glass homogenizer. To the homogenate was added perchloric acid (final concentration of 5%) followed by three freeze-thaw cycles and sonication on ice (4 cycles, high, 10 sec "on", 1 min "off"). The sample was then centrifuged at 1,811 g for 30 minutes at 4°C. The supernatant was neutralized using 50% KOH and recentrifuged. The residue was discarded and the supernatant was dried in a speedvac and stored at -20°C. The samples were reconstituted for NMR experiments in D_2_O containing DSS at 0.01%.

### ^1^H NMR spectroscopy

#### Urine spectra

1D^ 1^H NMR spectra of the urine samples were acquired on AVANCE 500 MHz Bruker spectrometer with broad-band inverse probe using DSS as an internal standard and D_2_O as the frequency lock at 300 K. The pulse sequence used included an excitation sculpting routine for the suppression of the water signal. 64 transients were collected into 8192 data points (TD) using a spectral width of 12.01 ppm resulting in an acquisition time of 0.68 s. A relaxation delay of 1 s was used between consecutive pulses leading to a repetition time of 1.68 s. The FIDs were subjected to exponential multiplication causing additional line-broadening of 0.2 Hz. A sine bell apodization function was also applied prior to Fourier transformation. In order to identify the metabolites, 2D NMR ^1^H-^1^H correlation spectroscopy (COSY) and total correlation spectroscopy (TOCSY) experiments were conducted on selected samples. In both these experiments, 32 transients per increment and 256 increments were collected in the indirect dimension. A QSINE function with 2048 and 1024 digital points were used for processing the 2D COSY. The TOCSY data was processed using SINE function. For both the datasets exponential multiplication of 0.20 and 0.30 Hz in the direct and indirect dimension respectively were used. In addition, a Gaussian multiplication of 0.1 Hz in the indirect dimension was applied for the TOCSY data.

#### Serum and brain spectra

1D^ 1^H NMR spectra of these samples were acquired on AVANCE 700 MHz Bruker spectrometer with broad-band inverse probe using DSS as an internal standard and D_2_O as the frequency lock at 300 K. The pulse sequence used included an excitation sculpting routine for the suppression of the water signal. 1024 transients were collected into 32,768 data points (TD) using a spectral width of 12.01 ppm resulting in an acquisition time of 1.94 s. A relaxation delay of 1 s was used between consecutive pulses leading to a repetition time of 2.94 s. The FIDs were subjected to exponential multiplication causing additional line-broadening of 0.2 Hz. A sine bell apodization function was also applied prior to Fourier transformation. In COSY experiments for these samples, 64 transients per increment and 256 increments were collected in the indirect dimension. A QSINE function with 2048 and 1024 digital points were used for processing. Exponential multiplication of 0.20 and 0.30 Hz in the direct and indirect dimension respectively was used. Online databases were used to acquire spectral information about relevant metabolites [22].

### Data reduction

Spectra were manually phased and baseline corrected. Spectral region of 0.5 to 9.5 ppm was segmented into frequency bins of 0.04 ppm for each spectrum and each bin was integrated using MestReC 4.7.0. The region corresponding to water and urea (4.5 to 6.5 ppm) was excluded during binning to avoid artifacts due to pre-saturation of water and the highly variable urea. Certain regions of the spectrum were found to contain peaks of high concentration with high variances across spectra/animals, which bore no correlation with infection. These regions were overestimated in PCA analyses masking potentially influential metabolites present in lower concentrations. Exclusion of these regions (3.25 to 3.46 (taurine), 2.82 to 2.89 (trimethylamine), 2.50 to 2.78 (citrate), 1.86 to 1.94 (acetate) ppm) from the initial analyses improved the quality of the models and allowed us to see differences arising due to infection more clearly. Such an observation has been made previously in other studies [17]. The resulting integrals were normalized to the total intensity of the spectrum to correct for inter-sample differences in concentration using an in-house developed program. For serum samples, the region from 1.26 to 1.38 ppm was removed from the data matrix prior to normalization because this lactate signal showed a pronounced increase post-infection that masked other changes.

### Statistical analyses

Statistical processing and visualization was achieved by SIMCA-P 12.0 (Umetrics AB, Umea, Sweden).

#### Urine

Data from different litters was modeled separately to avoid inter-litter variations. The spectral changes observed in litters 1 & 2 were however, comparable. PCA and OPLS-DA models were set up in three ways: (1) using data for all six mice from a litter (male and female); (2) using data from three mice of the same sex from a single litter; (3) modifying model 2 and using only the following data points (3a) days -2 to 0 and 1 to 4 (3b) days -2 to 0 and 5 to 8 (3c) days -2 to 0 and 9 to ~13. Models of type (1) establish differences between male and female samples (2) establish the extent of separation between uninfected and infected samples within the same sex cohort and (3) establish whether there is an increasing metabolic change with the progression of the disease.

#### Serum and brain

Data from serum and brain were treated separately. In each case, PCA and OPLS-DA models were set up in the following ways: (1) using data from all 24 animals (2) using data from control males and day 5 post-infection infected males (3) using data from control males and day 13 post-infection infected males (4) using data from control females and day 5 post-infection infected females (5) using data from -control females and day 13 post-infection infected females.

In OPLS-DA models within the same sex cohort, uninfected samples were treated as class 1 and infected samples as class 2. In other models males were treated as class 1 and females as class 2. All spectral data was Pareto scaled. One predictive component and all significant orthogonal components were calculated in each model. For all the PCA and OPLS-DA models, two-dimensional scores plots aided in visualization and representation of the sample clustering. In some cases to visualize a scores plot, a non-significant orthogonal component had to be calculated. R^2^X (cum) and Q^2 ^(cum) values from each model indicated its quality. They represent goodness of fit and predictability of the model respectively. S-plots along with loadings and variable importance plots of OPLS-DA models were used to interpret results and arrive at the spectral bins causing differences between classes.

### Peak integration

Following the identification of spectral regions and metabolites of interest, individual 1D NMR spectra were integrated for specific peaks using TOPSPIN 2.1 software. The peak intensities in each spectrum was calculated relative to the peak height of DSS and normalized to the total intensity of the spectrum (urine) or the wet weight of the tissue (serum and brain). Comparisons for significance were made using Student's t-test.

## Results

### Unsupervised clustering in urine data

Since urine is the most non-invasive sample, we tracked urinary changes of the same animals pre- and post-infection, with average 24-hour samples collected from each animal over a total of 20 to 22 days (Figure 1A). These mice were kept individually in metabolic cages and thus, uninfected mice were also maintained to ascertain that the stress of stay in metabolic cages for a prolonged period did not contribute to metabolic imbalance. The parasitaemia of infected mice was monitored throughout this period (Figure 1B). For an overview of our dataset, to determine its quality and to identify samples that are serious outliers, first Principle Component Analysis was performed. This established in an unsupervised manner, whether *Plasmodium *infection changed the metabolism in the host. It showed that data-points corresponding to pre- and post-infection days were differentiable from each other when data for a single animal was visualized (Figure 1C), indicating that the metabolite profile of the animal changed with infection. When data from multiple animals was combined in PCA scores, the post-infection changes were more difficult to visualize [see additional file 1]. All subsequent analyses were carried out by *a priori *assignment of samples into classes of either uninfected and infected or male and female. This was done by Orthogonal Partial Least Square Discriminant Analysis (OPLS-DA).

### Difference in male-female urinary metabolic profiles

**Figure 3:**
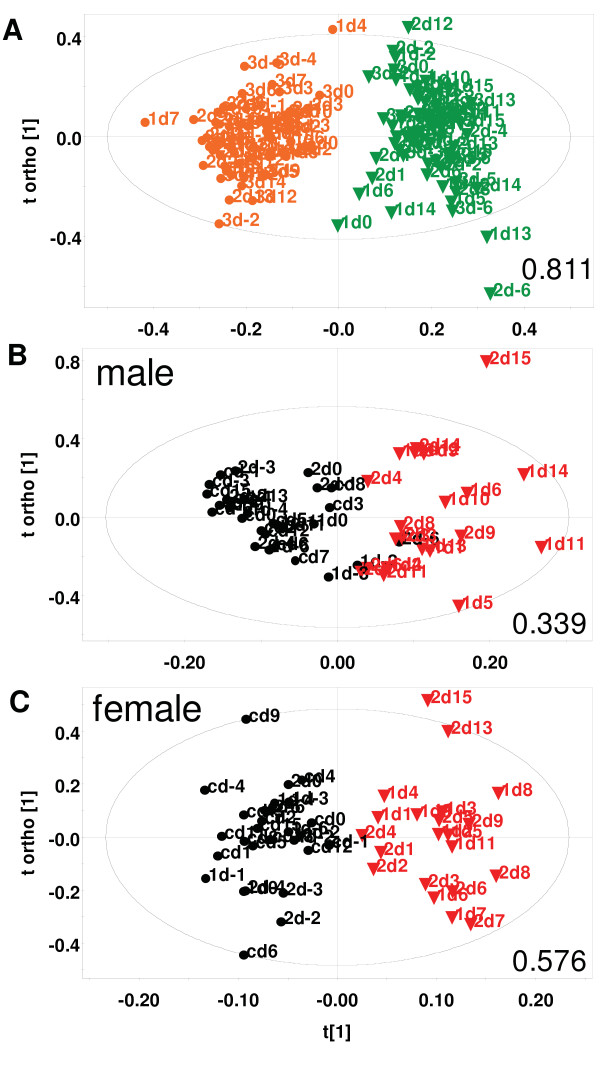
**^1^H NMR based OPLS-DA scores of urine of mice with/without infection with *Plasmodium berghei ANKA***. A. The model is from data of 6 uninfected mice of a litter (3 male, 3 female). Each point represents a sample corresponding to a mouse for the day indicated. The first number in the label indicates the mouse (1, 2 or 3); orange circle = male, green triangle = female. R^2^X (cum), Q^2^(cum) = 0.645, 0.811. B and C. Data from 3 mice (2 infected on day 7) of a litter monitored over 22 days, has been modeled in each case. Each point represents a mouse corresponding to the day indicated. The first number in the label indicates the mouse (1 = inf1, 2 = inf2, c = control). Black circle = uninfected, Red triangle = infected. R^2^X (cum), Q^2^(cum) are B. 0.632, 0.339, C. 0.73, 0.576. The ellipse is a 95% Hotelling's T^2 ^ellipse.

The initial examination of urine spectra from one batch of uninfected (normal) mice showed very high male-female class segregation (Q^2^(cum), an indicator of the predictability of the model = 0.811, Figure 3A). This difference remained large even in the two batches where malaria infection was introduced (Q^2^(cum) = 0.840, 0.922) as also when only late stage infected samples were considered [see additional file 2]. This confirmed that male and female urinary metabolic profiles are inherently different in mice. Such metabolically distinct profiles of urine samples and the contributing metabolites have been reported in humans and Hans Wistar rats [23,24]. To investigate malaria infection related changes specifically therefore, data from male and female mice were analyzed separately.

### Changes in urinary metabolic profile with progression of disease

Uninfected and infected urine samples were defined as two classes in the two sets of experiments. They were distinct (Q^2^(cum) = 0.321, 0.339 for males; 0.528, 0.576 for females) from each other in every litter (Figure 3B and 3C). The same distinction was not seen between pre- and post-day 0 data points in the batch where no mice were infected (Q^2^(cum) = -0.022 for males; 0.161 for females), [see additional file 3]. Both males and females showed a significant shift from control samples of the same sex when they were infected. This established that the infection causes the metabolic profile of infected animals to change considerably. There was no significant change in the profile if the mice were not injected with parasite.

**Figure 4:**
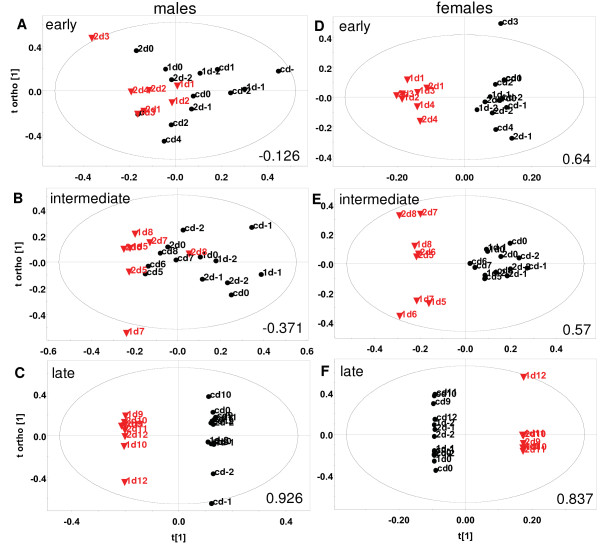
**Representative OPLS-DA scores of ^1^H NMR spectra of mouse urine showing post-infection temporal changes**. A-C male. D-F female. Data-points of days -2 to 0 (pre-infection) for 3 mice of a litter have been compared with A, D: days 1 to 4 (early stage infection); B, E: days 5 to 8 (intermediate stage infection) and C, F: days 9 to ~13 (late stage infection). Each point represents a sample corresponding to the day indicated. The first number in the label indicates the mouse. 1 = inf1, 2 = inf2, c = control. Black circle = uninfected, Red triangle = infected. The ellipse is a 95% Hotelling's T^2 ^ellipse. For A and B non-significant orthogonal components had to be calculated. R^2^X(cum), Q^2^(cum) = A. 0.569, -0.126; B. 0.531, -0.371; C. 0.925, 0.926; D. 0.752, 0.64; E. 0.558, 0.57; F. 0.911, 0.837.

Next, statistical models (3a), (3b) and (3c) were constructed (see Methods) which captured the early (days 1 to 4), intermediate (days 5 to 8) and late (days 9 to ~13) stages of infection respectively. These were used to address two questions. One, whether these metabolic profile changes became more pronounced when specific stages of infection were considered (i.e. whether Q^2^(cum) values improved). Secondly, to ask if the urinary profiles of mice became increasingly different as the infection progressed. Figure 4 shows the typical scores plots of these models. It was observed that in males, the change was negligible in early and intermediate stages (Q^2^(cum) = -0.126, 0.058 early; -0.371, -0.163 intermediate) as seen in Figure 4A and 4B. One mouse was found to behave as an outlier in litter 2 at the early stage of infection and was therefore excluded during the calculation of that model. In females however, the metabolic change was apparent within the first few days post-infection (Q^2^(cum) = 0.640, 0.227 early; 0.570, 0.574 intermediate), as seen in panels D and E of Figure 4. The parasitaemia levels of male and female mice in all stages remained comparable (Figure 1B). In the late stage males showed high differences from uninfected data with Q^2^(cum) values of 0.926 and 0.789 (Figure 4C). However females showed a variable trend (Q^2^(cum) = 0.837 and 0.335). Data from one of the litters is plotted in each panel of Figure 4. Beyond day 13 post-infection, the parasitaemia rises beyond 30% and the mice die within four to six days.

### Perturbed metabolites: urine

**Figure 5:**
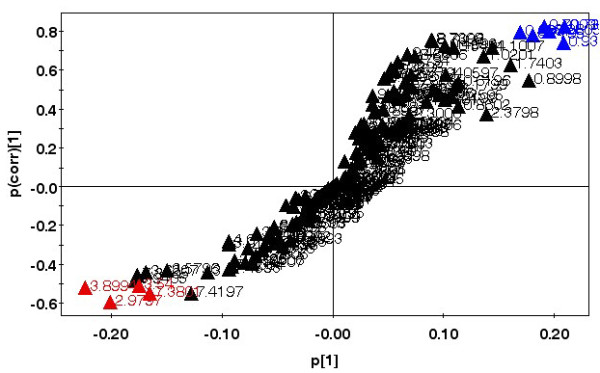
**Representative OPLS-DA S-plot showing relative contribution of bins to clustering of uninfected and infected classes**. This plot corresponds to scores plot Figure 4C. Red - important bins increased post-infection, Blue - important bins decreased post-infection. Bins with high correlation with class separation (>0.5 for increased, >0.75 for decreased) and high magnitude (>0.15) were selected for further analysis.

In addition to the differences shown in temporal response to the parasite, male and female mice also showed distinct differences in the metabolites that were perturbed with infection in urine upon comparison with control samples of the same sex. Figure 5 shows a representative S plot (correlation of spectral bins with infected-uninfected class separation) corresponding to the scores plot of Figure 4C where urine samples from late stage infected males were compared with control males. Such plots in conjugation with loadings and variable importance plots were used to identify relevant spectral regions contributing to the class segregation. A list of the metabolites that showed changes in their levels is given in Table 1. The identification of the metabolites was based on reported chemical shifts and 2D COSY experiments. The regions of the spectrum that change were very different between the sexes, excepting one commonality. The bin at 4.1 ppm which contained lactic acid was increased in both cases. This was the most predominant change observed in females when the late stage of infection was considered, but not in males. The region of 1.06 ppm showing an enhancement in females is likely to contain signals from increased levels of certain steroids, but these occur in very low concentrations and their presence could not be confirmed in the spectra.

**Table 1:** Urinary metabolites perturbed in mice upon infection with *Plasmodium berghei ANKA*

Increased Compound	Chemical shift	VIP value	Loading	Decreased Compound	Chemical shift	VIP value	Loading
**Females early stage**							
Ureidopropionate	2.34	3.02	+0.228	Carnitine	2.40	2.46	-0.179
**Females late stage**							
Lactate	4.10	2.89	+0.325	Carnitine	2.40	2.74	-0.318
Unidentified	1.06	1.63	+0.188	Unidentified	6.98	1.53	-0.174
Asparagine, DMG	2.90	1.60	+0.177				
**Males late stage**							
Creatine	3.90	3.34	+0.409	Unidentified	0.74	2.35	-0.255
Kynurenic acid	7.38	1.79	+0.205	Unidentified	0.70	2.14	-0.253
Phenylacetylglycine	7.38	1.79	+0.205	Unidentified	0.98	2.17	-0.245
GABA, Glutathione, Histamine, Oxoglutaric acid	3.00	2.20	+0.184	Lysine, Caprylate, Valerate, Alanine, Ethylmethyl acetate	1.5	1.25	-0.137
Lactate	4.10	1.60	+0.181				
Quinolinic acid	7.42	0.94	+0.117				

Females showed a shift in metabolism in the early stage of infection, which was marked by an increase of ureidopropionic acid and a decrease of carnitine. Reduction in urine carnitine levels in females at the early infection time-point was confirmed by individual peak integration (p = 0.015). These levels remained low even in the late stage (p = 0.004). However these trends were not observed in males.

Individual peak integration also revealed other distinctions in post-infection male and female urinary profiles that were not apparent from OPLS-DA models. It was observed that in stage-matched infected animals, the peak belonging to asparagine and DMG showed different trends in male and female mice [see additional file 4]. In females, the levels rose in the early stage and became comparable to controls in the late stage, whereas the levels continuously fell over the period of infection in male mice. The decrease in alanine and glycerol levels was significantly more in males, in both early and late stages of infection [see additional file 4]. Kynurenic acid levels are reduced in both males and females in the early stage of infection, however the decrease in levels is marginally larger in males (p = 0.08).

### Difference in male-female serum and brain metabolic profiles

Figure 2B shows the OPLS-DA scores of ^1^H NMR spectra of sera and brain extracts of the male and female mice. No male-female class segregation was observed (Q^2^(cum) = -0.156 serum; -0.278 brain). This holds regardless of the presence or absence of parasitic infection. This was contrary to our observation based on ^1^H NMR spectra of urine samples. This established that while in the urine, metabolites were excreted differentially; overall serum and brain metabolisms were not significantly divergent in the two sexes.

### Changes in serum and brain metabolic profiles with progression of disease

In order to investigate if differential temporal changes occurred in blood and brain, sera and brain samples were collected at early stage (day 5 post-infection) and late stage (day 13 post-infection) from animals. OPLS-DA scores of ^1^H NMR spectra of these tissue extracts of mice with progression of infection are shown in Figure 2C and 2D. Serum profiles showed a temporal trend that was different from the urine. When data from early infection stage was compared with uninfected controls, the segregation was high in males (Q^2^(cum) = 0.531) and negligible in females (Q^2^(cum) = -0.241). The brain profiles were unperturbed in the early stages (Q^2^(cum) = 0.08 males, -0.199 females). In the late stage, the serum and brain profiles changed drastically in both males (Q^2^(cum) = 0.983 serum, 0.991 brain) and females (Q^2^(cum) = 0.977 serum, 0.933 brain). A summary of all changes in terms of Q^2^(cum) is provided in additional file 5.

### Perturbed metabolites: serum and brain

**Figure 6:**
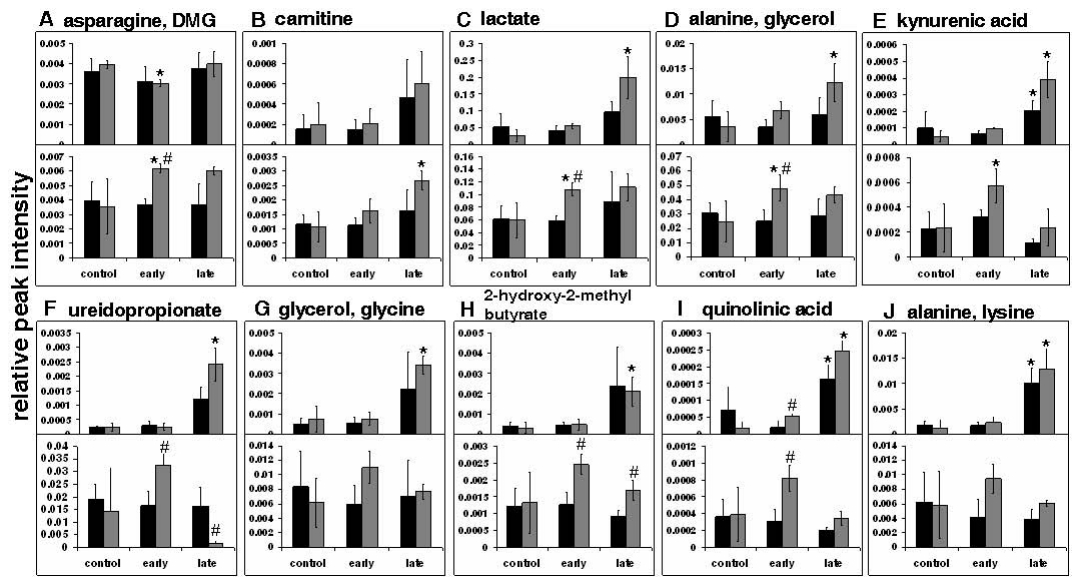
**Perturbed metabolite levels in mouse serum and brain at early and late stage malarial infection**. All peak intensities have been calculated relative to the peak height of 0.132 mg/ml DSS present in every sample. Panels showing two metabolites represent overlapping ^1^H NMR resonances. Black = males, grey = females. * indicates p < 0.05 in comparison to the uninfected control of the same sex. # indicates p < 0.05 in comparison to the males of the same infection time-point. S = serum, B = brain.

Serum and brain profiles, like those of urine, showed sex-specific differences in metabolites that were perturbed with infection. The metabolites identified from urine, serum and brain OPLS-DA modeling were further investigated by specific peak integration of individual spectra to determine changes in their levels over disease progression and between sexes in the various tissues. An overview of confirmed increased and decreased metabolites in sera and brain extracts are shown in additional file 6. Metabolites identified by OPLS-DA in serum and brain showed high overlap with those identified in urine with the exception of certain aromatic signals which appeared more prominently in urine. All significant trends for sera and brain are shown in Figure 6 and are mentioned below.

As is expected in the late stages of a lethal *Plasmodium *infection, there were significant alterations in the serum levels of several metabolites. The levels of kynurenic acid, quinolinic acid and alanine and lysine were significantly increased in the sera of both male and female mice (Figure 6E, I and 6J). Other changes occurred only in female mice, as shown for lactate, alanine and glycerol, ureidopropionic acid, glycerol and glycine, and 2-hydroxy-2-methyl-butyric acid (Figure 6C, F, G and 6H). In the brain, carnitine levels were found to be significantly higher in females at this stage (Figure 6B).

The changes in metabolite levels indicated by OPLS-DA analysis in sera of male mice at the early stage of infection did not show significant trends when individual peaks were integrated. Significant early changes in metabolite levels were seen predominantly in female mice (Figure 6). In serum extracts, asparagine and DMG levels were significantly reduced (Figure 6A). In extracts from the brain, levels of lactate, asparagine and DMG, alanine and glycerol, and kynurenic acid were increased only in females in the early stage of infection (Figure 6A, C, D and 6E).

### Perturbed metabolites: male-female differences during disease progression in serum and brain

Direct comparisons of metabolite levels in male and female mice at both post-infection stages of infection were also made. Serum levels of quinolinic acid were higher in females when compared to males in the early stage of infection (Figure 6I). However in brain extracts from the same animals, this trend was observed for several metabolites namely lactate, asparagine and DMG, alanine and glycerol, quinolinic acid, ureidopropionic acid and 2-hydroxy-2-methyl-butyric acid during early infection (Figure 6A, C, D, F, H and 6I). In the late stage of infection, females had lower ureidopropionic acid and higher 2-hydroxy-2-methyl-butyric acid when compared to males.

## Discussion

The large-scale metabolic phenotyping of malaria-infected tissues and body fluids carried out in this study brings into focus several aspects of the host response to the parasite. This data strongly indicates that male and female animals respond to the infection in different ways. Urine profiles of female mice were altered much earlier as compared to males during disease progression. Serum profiles on the other hand, showed global changes in male mice in early infection but not in females. Overall metabolism changed drastically in the late stages in both sexes. However, when levels of specific metabolites were assessed, changes occurred in the early stages in all three tissues/body fluids investigated, and the data indicates that majority of these are seen in female mice. The metabolites that showed such alterations included carnitine, ureidopropionate, asparagine and dimethylglycine, lactate, quinolinic acid, kynurenic acid, 2-hydroxy-2-methylbutyrate, alanine and glycerol. They are indicative of perturbations in multiple pathways including glycolysis, lipid metabolism, tryptophan metabolism and uracil degradation. Since the responses along these pathways are more prominent in female mice, these results are important for understanding the differential pathophysiology of malaria in the two sexes.

Inherent sex differences in urine metabolites have been documented earlier in rats as well as in humans [23,24]. The high values of Q^2^(cum) in the data reported here (0.811) and that in humans (0.47) when male versus female urine samples are compared, suggests a very significant divergence in their metabolism [see additional file 5], [24]. It was also observed that removal of up to 50% spectral bins from the data set in OPLS-DA analysis failed to change the male-female segregation considerably (Q^2^(cum) = 0.729). However such sex-specific divergence did not exist in serum and brain tissue metabolites (Figure 5B, additional file 5). In most murine studies same sex animals are examined for disease related changes [17,20,25]. This is the first report of an elaborate comparison of sex related disparity in metabolic changes in response to a pathogen.

Urine samples represent the global metabolic state of the host and are non-invasively obtained. Multivariate analyses of ^1^H NMR spectra of urine enabled us to visualize temporal changes in the host response and identify the sexual dimorphism therein. Further, when serum and brain profiles of infected mice were investigated, brain metabolism was found to be unaltered in the early stages when the complete metabolite profile is considered; indicating that brain tissue is largely uninfluenced by the parasite at the onset of malaria. The observation that serum profiles changed early in males, while their urine profiles did not, raises the possibility that female hosts are able to regulate their metabolism to maintain homeostasis in the blood.

The increase in lactic acid reported in this study is expected since *Plasmodium *infection is known to increase glycolytic rate in RBCs [26-28]. Additionally, a sexual dimorphism is also observed in serum and brain levels of lactate (Figure 6C). Since parasitaemia levels are not significantly different between the two sexes, it is plausible that the change in lactate levels in females is not solely due to increased glucose utilization by the parasite and may reflect enhanced glycolysis in the host. Up-regulation of glycolysis would also lead to enhanced transamination of pyruvate to alanine. Alanine clearance and alanine to lactate ratio have been correlated with the severity of clinical malaria [29]. In the data reported here, the variations in alanine levels parallel those of lactate, suggesting that these changes may be linked to their common substrate pyruvate (Figure 6D). Alanine has been reported to be up-regulated in brain tissue of female BALB/c mice infected with *Plasmodium berghei *ANKA [3].

An imbalance of amino acid metabolism in the disease process manifests in altered levels of several amino acids [3,8]. In this study, alanine, lysine, glycine and asparagine levels are perturbed during disease progression. Lysine levels are increased in late stage male and female sera. A previous metabolomic study in malaria has reported prominent enhancement of pipecolic acid, a neuromodulator formed from the degradation of lysine [30]. This molecule was not observed in the experiments of this report possibly due to the difference in strain and age of animals used. Of particular interest is the observed trend of asparagine levels in females during early phase of the disease (increase in urine and brain, decrease in serum). *Plasmodium *species incorporate disproportionately large amount of asparagine in its proteome in the ubiquitous low complexity regions [31]. However, it is unclear how this might impact the sex-specific manner in which asparagine levels change.

Kynurenic acid (KA) and quinolinic acid (QA) were observed to be enhanced in urine in male infected mice (Table 1). The variation exhibited a pronounced sexual dimorphism during early infection when sera and brain were considered (Figure 6E). Changes in KA and QA have been implicated in the pathogenesis of cerebral malaria. They are involved in an oxidative metabolic pathway that converts tryptophan into kynurenine via IFN-γ activated indoleamine 2, 3-dioxygenase (IDO). Kynurenine is further converted into KA and QA via two divergent pathways [32]. The ratio of QA to KA is reported to be increased in the brain of mice with cerebral malaria (CM), but not in non-cerebral malaria (NCM) [32]. Mice with CM have also been found to survive longer when this pathway is inhibited [33]. In addition, IDO activity has also been reported to be up-regulated in the vascular endothelium of several tissues of mice infected with *P. berghei *in a previous study [34]. The set up used here with BALB/c mice and *P. berghei *is an NCM model. The results identify mis-regulation along the kynurenine pathway in urine, sera and brain. Furthermore, the early stage alterations are only in females and not in males.

Ureidopropionate is a known intermediate in the breakdown of uracil to β-alanine. Its accumulation in body fluids is indicative of a deficiency of ureidopropionase and represents a severe form of propionic aciduria. Ureidopropionate is an endogenous neurotoxin manifesting in severe neuropathology of the grey and the white matter [35]. The observed variation in levels of this metabolite is suggestive of the aforementioned condition. It remains to be established how the presence of the parasite influences this pathway selectively in females (Figure 6F). Variations in 2-hydroxy-2-methylbutyrate also exhibited differences in males and females (Figure 6H). In humans, 2-hydroxy-2-methylbutyrate is found in urine of patients with 2-hydroxyglutaric aciduria [36]. This condition is accompanied by cerebellar atrophy and subcortical leukoencephalopathy. The observed increase of this metabolite in female brain suggests that females may be (at least transiently) more prone to such neural degeneration early in the presence of the parasite.

Glycerol levels showed an upward trend that is significant in the sera of late stage female mice (Figure 6D and 6G). Fasting glycerol concentrations and turn-over of infused glycerol in malaria patients has been reported to be higher than in patients recovering from the disease [37]. OPLS-DA results in this paper also suggested an incremental trend in long chain fatty acid concentrations in serum [see additional file 5] and urine. Oxidation of these fatty acids would entail the recruitment of carnitine. The decrease in carnitine levels in urine observed solely in females suggests a selectively higher retention of this metabolite for cellular β-oxidation of fatty acids. This is in contrast to normal resting situation in humans, where elevated levels of carnitine and acetylcarnitine in males relative to females were observed [24]. Dimethylglycine (DMG) is an intermediate derived from choline, which may be acquired from the diet or through catabolism of phospholipids [38,39]. Higher DMG has been observed previously in normal female Wistar rats as compared to males [23]. Here an early increase of DMG in brain tissue, a decrease in sera and a transient rise in urine levels was observed in females as opposed to males (Figure 6A). Accumulation of DMG in plasma has been reported to be an indicator of chronic renal failure [40]. It remains to be ascertained how the sexual dimorphism observed in DMG level variation may have a bearing on renal function.

Metabolic profiling of several diseases such as sepsis, those caused by microbial pathogens *Streptococcus, Staphylococcus *and by parasites, such as *Trypanosoma *and *Schistosoma *has been reported [17,20,25,41]. Lactate and alanine were observed to be altered in sepsis [41], *Trypanosoma *[20] and *Schistosoma *[17] infections. However, specific alterations reported here in the levels of carnitine, asparagine, KA, QA and ureidopropionate appear to be unique to malarial infection. The pathways involving these metabolites, specifically those in early responses may determine the specificity of malaria pathogenesis and lead to molecular predictors of disease progression.

Clinically many parasitic diseases have been reported to affect males and females differentially [42,43]. Males have been shown to be more susceptible to *Plasmodium, Trypanosoma, Giardia and Leishmania major*, while females are more susceptible to *Leishmania donovani, Trichomonas vaginalis *and *Toxoplasma gondii *[44]. In humans, although no sexual dimorphism is observed in most malaria endemic areas, the intensity of infection is reported to be higher for males [45]. Studies in adult mice, exposed for the first time to malaria parasites, have established a distinct sex-bias with higher susceptibility in male mice [46,47]. Through a transcriptomic study, some of the observed differences have been attributed to differential immune and haematopoietic responses, perhaps caused by differential hormonal regulation [47]. The demonstration here of early sensitization of females to *Plasmodium *infection establishes sexual dimorphism in systemic metabolic response of mice to malaria. It is unclear presently how such responses may be modulated in the host. The data from the present study provide candidate metabolites implicating pathways that differ between the sexes during disease progression. An understanding of early events would help towards the elucidation of mechanisms contributing to sexual dimorphism and severity in malaria.

## Abbreviations

NMR: Nuclear Magnetic Resonance; PCA: Principle Component Analysis; OPLS-DA: Orthogonal Partial Least Square Discriminant Analysis; CM: Cerebral Malaria; NCM: Non-Cerebral Malaria; GABA: γ-aminobutyric acid; IFN γ: Interferon gamma; KA: Kynurenic acid; QA: Quinolinic acid; DMG: Dimethylglycine.

## Competing interests

The authors declare that they have no competing interests.

## Authors' contributions

AB carried out all animal experiments, tissue extractions, NMR spectroscopy experiments, data processing and multivariate analyses as well as drafted the manuscript. MR standardized and performed the experiments for analyses of urinary metabolomic profiles in mice. SS conceived the study, helped design experiments, interpret the data and draft the manuscript. HMS conceived the study, helped design experiments, acquire and interpret NMR data and draft the manuscript. All authors read and approved the final manuscript.

## Supplementary Material

Additional file 1 **PCA scores plots of ^1^H NMR spectra of urine samples from three mice of a litter**. Post-infection changes in urinary profiles of A. male and B. female mice from a litter as visualized in unsupervised PCA scores plots. Black circle = uninfected, Red triangle = infected. Click here for file

Additional file 2 **OPLS-DA scores plots showing male-female distinction in late stage infected urine samples**. Sex-related differences can be visualized in urine samples from late stage infected male and female mice in these OPLS-DA scores plots. Orange circle = male, Green triangle = female. Q^2^(cum) = 0.838. Click here for file

Additional file 3 **OPLS-DA scores plots showing pre- and post-day 0 urine samples when no parasite was introduced**. In an experiment where no parasite was introduced on day 0, the class separation between pre- and post-day 0 in A. male and B. female urine samples was poor. Black circle = pre-day 0, Red triangle = post day 0. Q^2^(cum) values are indicated on the plots. Click here for file

Additional file 4 **Perturbed metabolite levels in mouse urine in early and late stage malarial infection**. Black = male, grey = female. The average peak intensity for the metabolite in control samples of the same sex was subtracted from individual peak intensities of infected animals at each stage of infection. The average change in intensity with respect to the same sex control is plotted here. p values for the comparison of changes in male and female animals at each stage are indicated. Click here for file

Additional file 5 **Summary of post-infection temporal changes and gender differences in metabolic profiles of different samples**. Values are in terms of Q^2^(cum) of OPLS-DA analysis, representing extent of separation between infected and uninfected or male and female populations for the tissue/body fluid indicated. Click here for file

Additional file 6 **Metabolites perturbed in sera and brain during infection**. OPLS-DA results showing global metabolite changes in A. males early stage serum, B. males late stage serum, C. females late stage serum, D. males late stage brain E. females late stage brain. Click here for file
