# Supplementary figures and images for: Alterations in urine, serum and brain metabolomic profiles exhibit sexual dimorphism during malaria disease progression
Source: Malar J. 2010 Apr 23;9:110. doi: 10.1186/1475-2875-9-110 (PMC2873523; doi:10.1186/1475-2875-9-110)

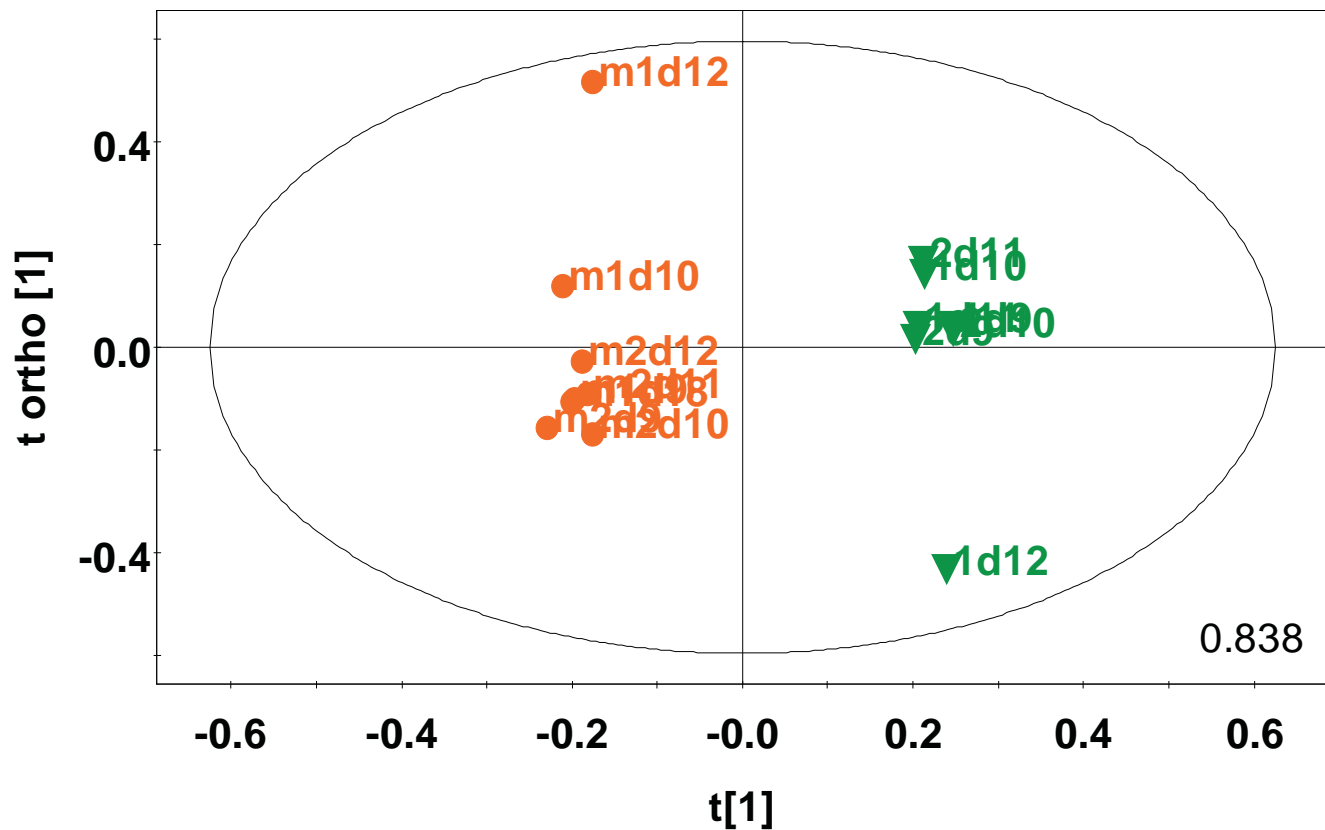

Supplement: Additional file 2 — OPLS-DA scores plots showing male-female distinction in late stage infected urine samples. Sex-related differences can be visualized in urine samples from late stage infected male and female mice in these OPLS-DA scores plots. Orange circle = male, Green triangle = female. Q2(cum) = 0.838. [file 1475-2875-9-110-S2.PDF]

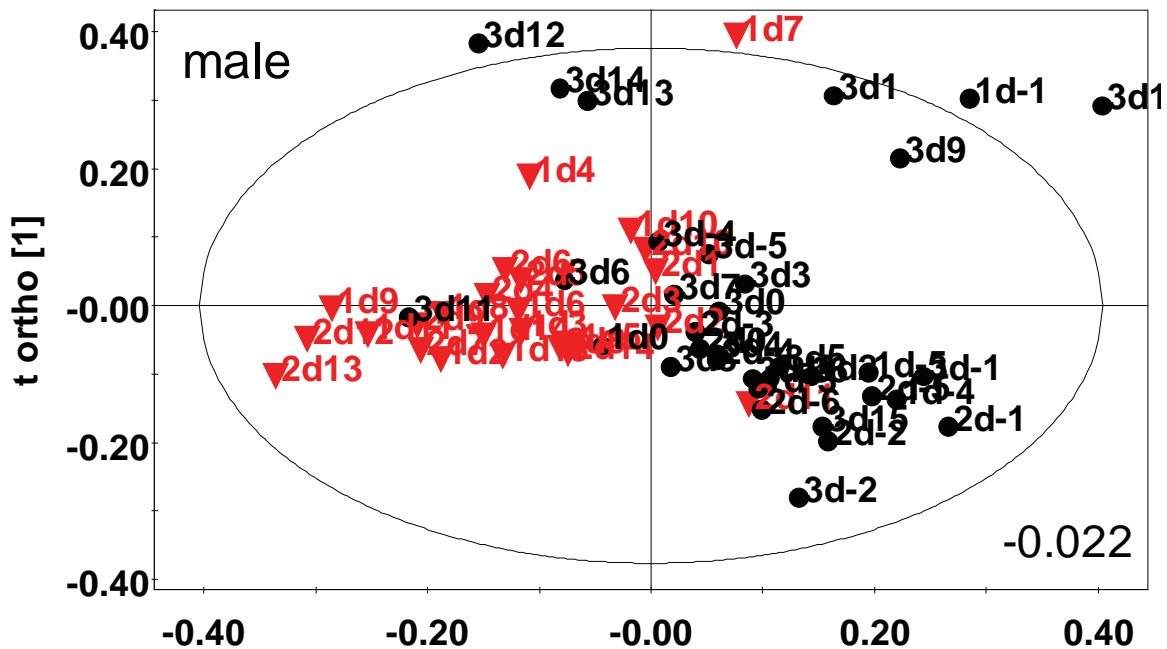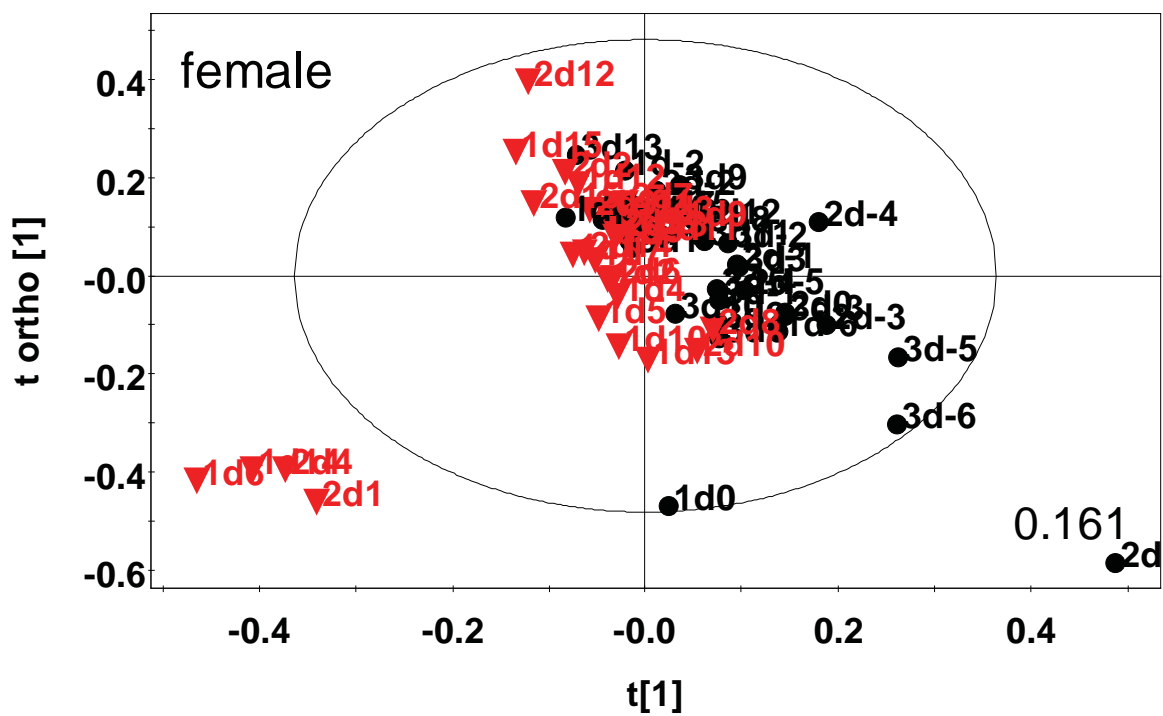

Supplement: Additional file 3 — OPLS-DA scores plots showing pre- and post-day 0 urine samples when no parasite was introduced. In an experiment where no parasite was introduced on day 0, the class separation between pre- and post-day 0 in A. male and B. female urine samples was poor. Black circle = pre-day 0, Red triangle = post day 0. Q2(cum) values are indicated on the plots. [file 1475-2875-9-110-S3.PDF]

### Asparagine, DMG

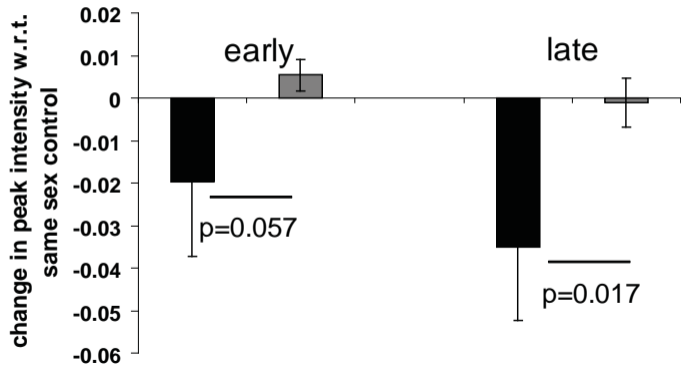

### Alanine, glycerol

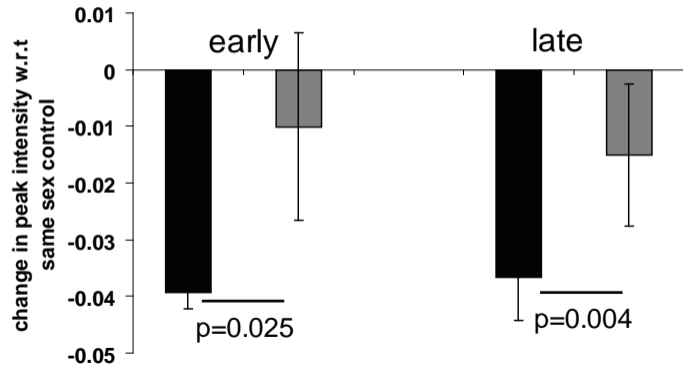

Supplement: Additional file 4 — Perturbed metabolite levels in mouse urine in early and late stage malarial infection. Black = male, grey = female. The average peak intensity for the metabolite in control samples of the same sex was subtracted from individual peak intensities of infected animals at each stage of infection. The average change in intensity with respect to the same sex control is plotted here. p values for the comparison of changes in male and female animals at each stage are indicated. [file 1475-2875-9-110-S4.PDF]
