# Supplementary material for: Alterations in urine, serum and brain metabolomic profiles exhibit sexual dimorphism during malaria disease progression
Source: Malar J. 2010 Apr 23;9:110. doi: 10.1186/1475-2875-9-110 (PMC2873523; doi:10.1186/1475-2875-9-110)
Supplement: Additional file 5 — Summary of post-infection temporal changes and gender differences in metabolic profiles of different samples. Values are in terms of Q2(cum) of OPLS-DA analysis, representing extent of separation between infected and uninfected or male and female populations for the tissue/body fluid indicated. [file 1475-2875-9-110-S5.DOC]

**Additional File 5. Summary of post-infection temporal changes and gender differences in metabolic profiles of different samples. Values are in terms of Q^2^**(cum), representing extent of separation between infected and uninfected or male and female populations. OPLS-DA analysis.

| *Sample* | *Male-female difference, Q^2^(cum)* | *Early stage, Q^2^(cum)* | | *Late stage, Q^2^(cum)* | |
| --- | --- | --- | --- | --- | --- |
|  |  | males | females | males | females |
| Urine | 0.811 | -0.126 | 0.64 | 0.926 | 0.837 |
| Serum | -0.156 | 0.53 | -0.241 | 0.983 | 0.977 |
| Brain | -0.278 | 0.08 | -0.199 | 0.991 | 0.933 |
