# Supplementary material for: Alterations in urine, serum and brain metabolomic profiles exhibit sexual dimorphism during malaria disease progression
Source: Malar J. 2010 Apr 23;9:110. doi: 10.1186/1475-2875-9-110 (PMC2873523; doi:10.1186/1475-2875-9-110)
Supplement: Additional file 6 — Metabolites perturbed in sera and brain during infection. OPLS-DA results showing global metabolite changes in A. males early stage serum, B. males late stage serum, C. females late stage serum, D. males late stage brain E. females late stage brain. [file 1475-2875-9-110-S6.DOC]

**Additional file 6. Metabolites perturbed during infection in A. males early stage serum B. males late stage serum C. females late stage serum D. males late stage brain E. females late stage brain**

**A.** males early stage, serum

| *Increased* | | | | *Decreased* | | | |
| --- | --- | --- | --- | --- | --- | --- | --- |
| *Compound* | *Chemical shift* | *VIP value* | *Loading* | *Compound* | *Chemical shift* | *VIP value* | *Loading* |
| Long chain fatty acids | 1.26 | 4.83 | +0.261 | Lactic acid | 4.1 | 2.96 | -0.237 |
| Unidentified | 3.18 | 4.64 | +0.361 | 3-methylcrotonylglycine, lactic acid | 4.14 | 2.86 | -0.235 |
| Choline, N-methylhydantoin, creatinine | 4.06 | 3.11 | +0.399 | Creatine | 3.9 | 2.68 | -0.214 |
| Unidentified | 0.58 | 2.30 | +0.189 |  |  |  |  |

**B.** males late stage, serum

| *Increased* | | | | *Decreased* | | | |
| --- | --- | --- | --- | --- | --- | --- | --- |
| *Compound* | *Chemical shift* | *VIP value* | *Loading* | *Compound* | *Chemical shift* | *VIP value* | *Loading* |
| Lactic acid | 4.1 | 3.84 | +0.311 | 2,3-butanediol | 3.7 | 2.70 | -0.219 |
| Alanine, lysine | 1.46 | 2.68 | +0.218 | Unidentified | 3.66 | 2.54 | -0.206 |
| 2-hydroxy-2-methylbutyric acid | 0.94 | 2.59 | +0.210 | Alanine | 3.74 | 2.38 | -0.193 |
| 2-ethylacrylic acid | 0.98 | 2.19 | +0.178 |  |  |  |  |
| Long chain fatty acids | 0.9 | 2.18 | +0.177 |  |  |  |  |

**C.** females late stage, serum

| *Increased* | | | | *Decreased* | | | |
| --- | --- | --- | --- | --- | --- | --- | --- |
| *Compound* | *Chemical shift* | *VIP value* | *Loading* | *Compound* | *Chemical shift* | *VIP value* | *Loading* |
| Lactic acid | 4.1 | 4.15 | +0.336 | Unidentified | 1.78 | 1.76 | -0.143 |
| Choline | 4.06 | 3.91 | +0.318 | Unidentified | 3.66 | 1.62 | -0.132 |
| Alanine, lysine | 1.46 | 3.68 | +0.298 | Alanine | 3.78 | 1.35 | -0.110 |
| 2-hydroxy-2-methylbutyric acid | 0.94 | 2.08 | +0.169 | Unidentified | 0.66 | 1.15 | -0.093 |
| Unidentified | 2.34 | 1.35 | +0.110 | Unidentified | 2.94 | 1.10 | -0.089 |
|  |  |  |  | Lactic acid | 4.14 | 1.08 | -0.087 |
|  |  |  |  | Unidentified | 3.1 | 0.54 | -0.044 |

**D.** males late stage, brain

| *Increased* | | | | *Decreased* | | | |
| --- | --- | --- | --- | --- | --- | --- | --- |
| *Compound* | *Chemical shift* | *VIP value* | *Loading* | *Compound* | *Chemical shift* | *VIP value* | *Loading* |
| Lactic acid | 1.3 | 5.42 | +0.436 | Glycerol | 3.54 | 4.42 | -0.355 |
| Unidentified | 1.98 | 3.23 | +0.259 | Glycerol, alanine | 3.78 | 2.65 | -0.213 |
| Carnitine | 2.42 | 3.01 | +0.242 | Unidentified | 2.34 | 2.27 | -0.182 |
| Choline | 4.02 | 2.58 | +0.207 | Unidentified | 3.02 | 2.17 | -0.174 |
| Unidentified | 2.98 | 2.44 | +0.196 | Unidentified | 3.94 | 1.97 | -0.158 |

**E.** females late stage, brain

| *Increased* | | | | *Decreased* | | | |
| --- | --- | --- | --- | --- | --- | --- | --- |
| *Compound* | *Chemical shift* | *VIP value* | *Loading* | *Compound* | *Chemical shift* | *VIP value* | *Loading* |
| Carnitine | 2.42 | 3.11 | 0.250 | Cystine | 3.18 | 3.18 | -0.340 |
| Glutamine | 2.14 | 1.64 | 0.132 | Unidentified | 3.02 | 3.02 | -0.232 |
| Putrescine, lysine | 1.74 | 1.60 | 0.094 | Choline | 4.06 | 4.06 | -0.221 |
| Lactic acid | 1.34 | 1.08 | 0.087 | Glycerol | 3.54 | 3.54 | -0.219 |
|  |  |  |  | Creatine | 3.9 | 3.9 | -0.208 |
