# Supplementary material for: Alterations in urine, serum and brain metabolomic profiles exhibit sexual dimorphism during malaria disease progression
Source: Malar J. 2010 Apr 23;9:110. doi: 10.1186/1475-2875-9-110 (PMC2873523; doi:10.1186/1475-2875-9-110)
Supplement: Additional file 1 — PCA scores plots of 1H NMR spectra of urine samples from three mice of a litter. Post-infection changes in urinary profiles of A. male and B. female mice from a litter as visualized in unsupervised PCA scores plots. Black circle = uninfected, Red triangle = infected. [file 1475-2875-9-110-S1.PDF]

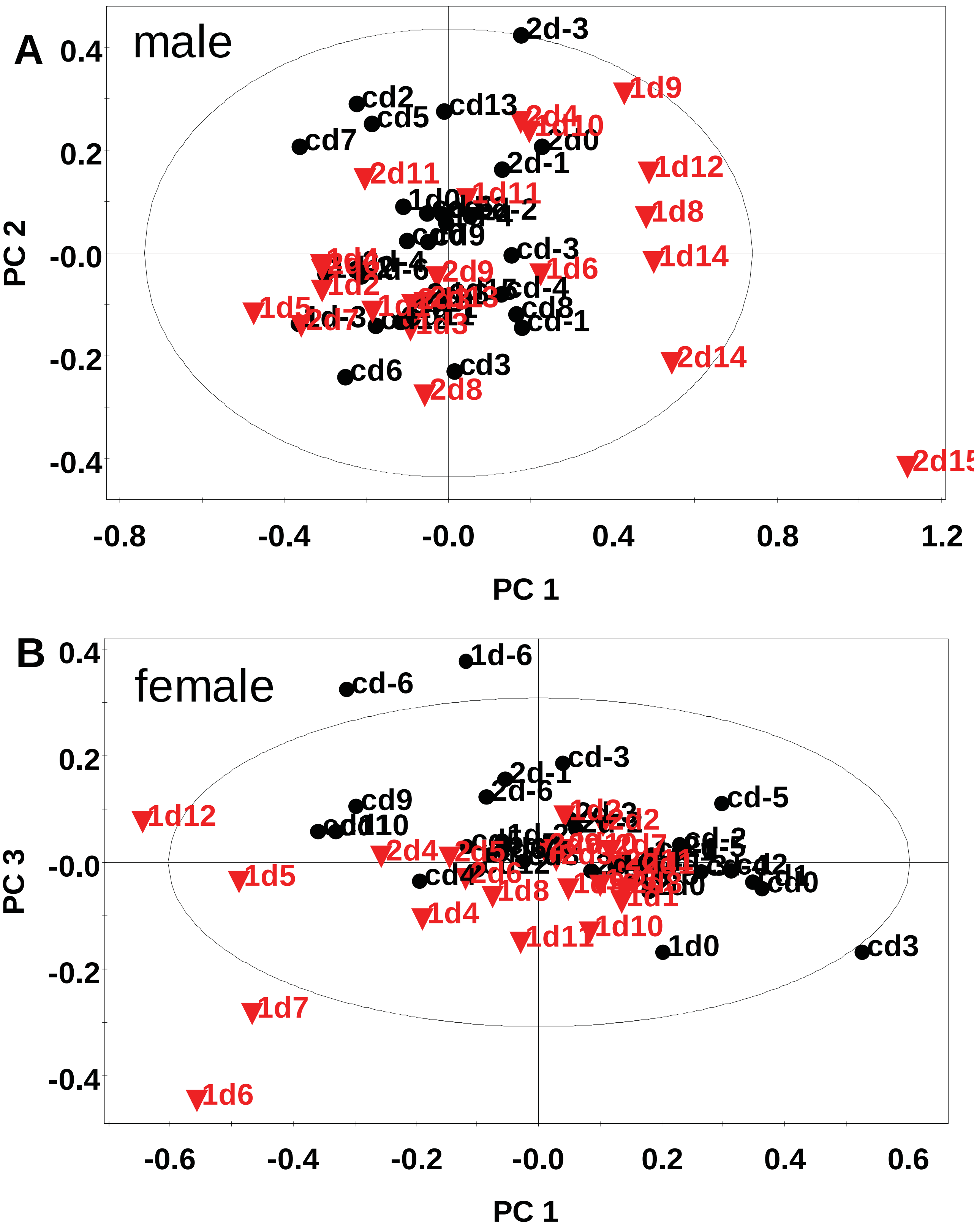

A
male
PC 2
PC 1
0.4
0.2
-0.0
-0.2
-0.4
-0.8
-0.4
-0.0
0.4
0.8
1.2
2d-3
1d9
cd2
cd13
cd5
2d4
1d10
cd7
2d0
2d-1
1d12
2d11
1d11
1d8
cd9
cd-3
1d14
1d6
2d9
cd-4
1d2
1d5
cd8
cd-1
1d3
2d7
2d14
cd6
cd3
2d8
B
female
PC 3
PC 1
0.4
0.2
-0.0
-0.2
-0.4
-0.6
-0.4
-0.2
-0.0
0.2
0.4
0.6
1d-6
cd-6
cd-3
2d-1
2d-6
cd9
cd-5
1d12
cd10
2d4
1d5
cd4
1d8
1d4
1d10
1d11
1d0
cd3
1d7
1d6
